# Stevens–Johnson syndrome/toxic epidermal necrolysis as the initial presentation of paraneoplastic anti-TIF1-γ dermatomyositis

**DOI:** 10.1177/2050313X251406472

**Published:** 2026-01-14

**Authors:** Ghassan Barnawi, Raquel Lazarowitz, Lara Al-Sabeh, Ammar Saed Aldien, Ivan V. Litvinov

**Affiliations:** 1 Division of Dermatology, McGill University, Montreal, QC, Canada; 2 Faculty of Medicine and Health Sciences, McGill University, Montreal, QC, Canada; 3 Faculty of Medicine, University of Montreal, QC, Canada; 4 Division of Dermatology, St. Mary’s Hospital Centre, Montreal, QC, Canada

**Keywords:** Stevens–Johnson syndrome, toxic epidermal necrolysis, dermatomyositis, cancer, DLBC-lymphoma

## Abstract

We report a rare case of Stevens–Johnson syndrome/toxic epidermal necrolysis as the initial manifestation of paraneoplastic dermatomyositis in a 50-year-old man subsequently diagnosed with diffuse large B cell lymphoma, in the absence of any identifiable drug exposure. The patient presented with periorbital edema, progressive dusky blistering eruption (Nikolsky sign positive), mucositis, and histopathology consistent with Stevens–Johnson syndrome/toxic epidermal necrolysis. Over the following weeks, he developed proximal muscle weakness, elevated creatine kinase, and cutaneous signs of dermatomyositis, including heliotrope rash and Gottron’s papules, with serologic confirmation via anti-TIF1-γ antibodies. Imaging revealed a hypermetabolic axillary mass, and excisional biopsy confirmed diffuse large B cell lymphoma. This case highlights a novel presentation of Stevens–Johnson syndrome/toxic epidermal necrolysis in the setting of autoimmune and paraneoplastic immune dysregulation. It underscores the importance of considering nondrug-induced triggers in Stevens–Johnson syndrome/toxic epidermal necrolysis.

## Introduction

Stevens–Johnson syndrome (SJS)/toxic epidermal necrolysis (TEN) is a severe mucocutaneous hypersensitivity reaction characterized by painful erythema, exanthematous eruption that evolves into epidermal detachment, mucosal erosions/ulcers, systemic symptoms and can progress to multiorgan involvement.^1^ The disease primarily occurs in response to a recent medication exposure, which induces a dysregulated immune response. Infections/viruses are also recognized as causes, particularly in pediatric patients.^1^ A higher incidence of SJS/TEN has been reported among cancer patients, though the role of malignancy-related immune dysregulation versus increased drug exposure remains unclear.^1^ Additionally, SJS/TEN-like eruptions have been described in autoimmune conditions such as systemic lupus erythematosus and dermatomyositis.^2–4^

We present a novel case of SJS/TEN in a 50-year-old patient, presenting as the initial manifestation of paraneoplastic dermatomyositis associated with newly diagnosed diffuse large B cell lymphoma (DLBCL). To our knowledge, this is the first reported case of SJS/TEN presenting as a paraneoplastic phenomenon in dermatomyositis, in the absence of any identifiable drug culprit.

## Case presentation

**Figure 1.**
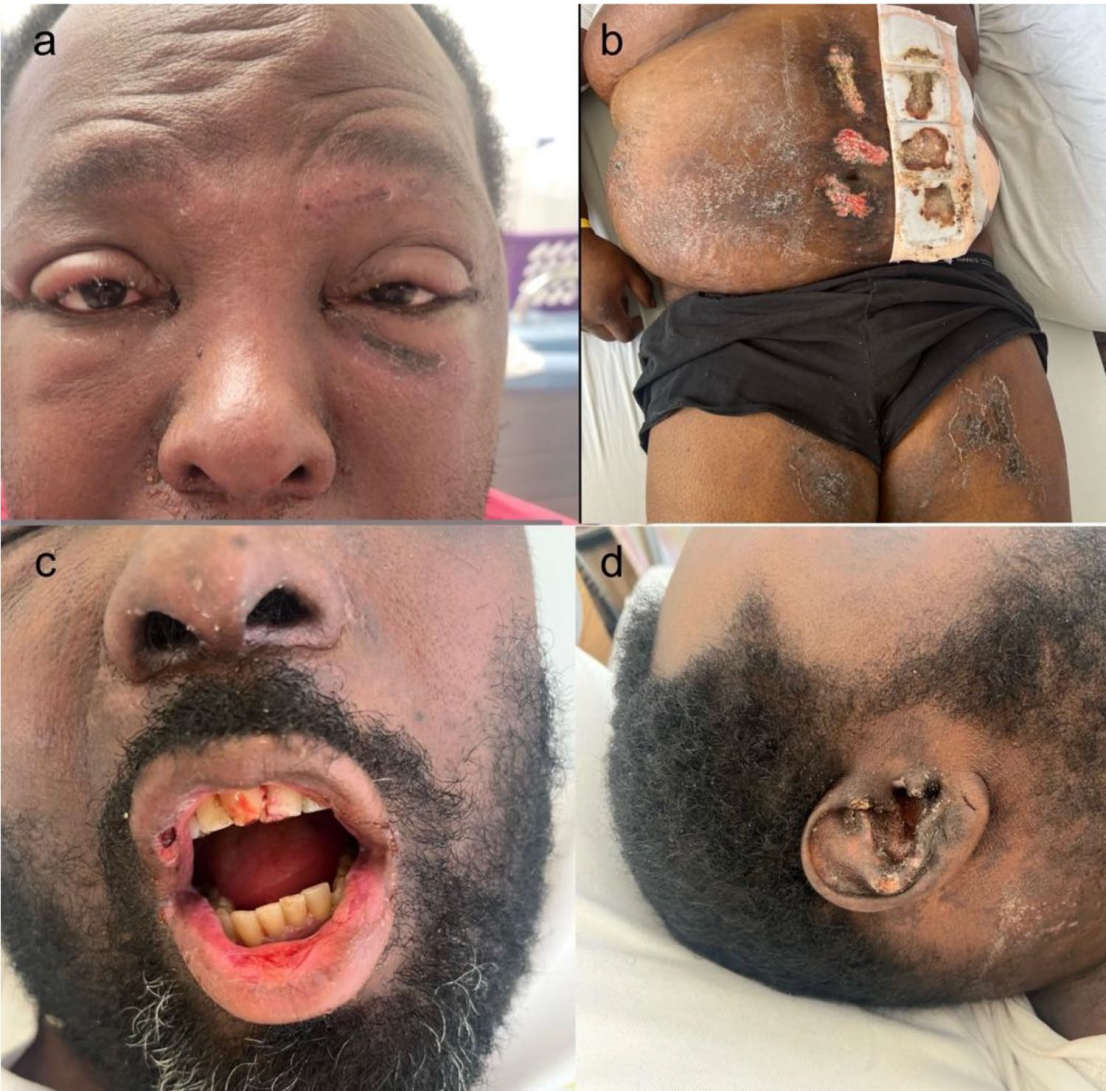
(a) Eyelid and periorbital edema with facial swelling. (b) Healing scalded-skin appearance on the trunk, consistent with re-epithelializing lesions. (c) Hemorrhagic mucosal erosions involving the lips and oral cavity. (d) Right ear mucosal erosions and facial swelling.

A 50-year-old Afro-Canadian male with a medical history of hypertension, type 2 diabetes mellitus, and dyslipidemia presented to the emergency department with a 5-day history of progressive periorbital, eyelid edema, and facial swelling (Figure 1(a)). This evolved gradually over the next 3 weeks into a dusky, painful, desquamating, and blistering rash that began on the chest and later spread to the face, trunk, and extremities, accompanied by erosive oral mucositis and ocular symptoms (Figure 1(b)–(d)). The patient has no history of autoimmune conditions, denied upper respiratory tract infection symptoms, or the use of any new medications in the preceding days to his presentation.

Two different skin biopsy samples were obtained and demonstrated full-thickness epidermal necrosis. No direct immunofluorescence was performed. A provisional diagnosis of SJS/TEN of unknown etiology was made. The patient was started on cyclosporine, etanercept, doxycycline, and supportive care for erosions, which resulted in clinical improvement of his disease.

**Figure 2.**
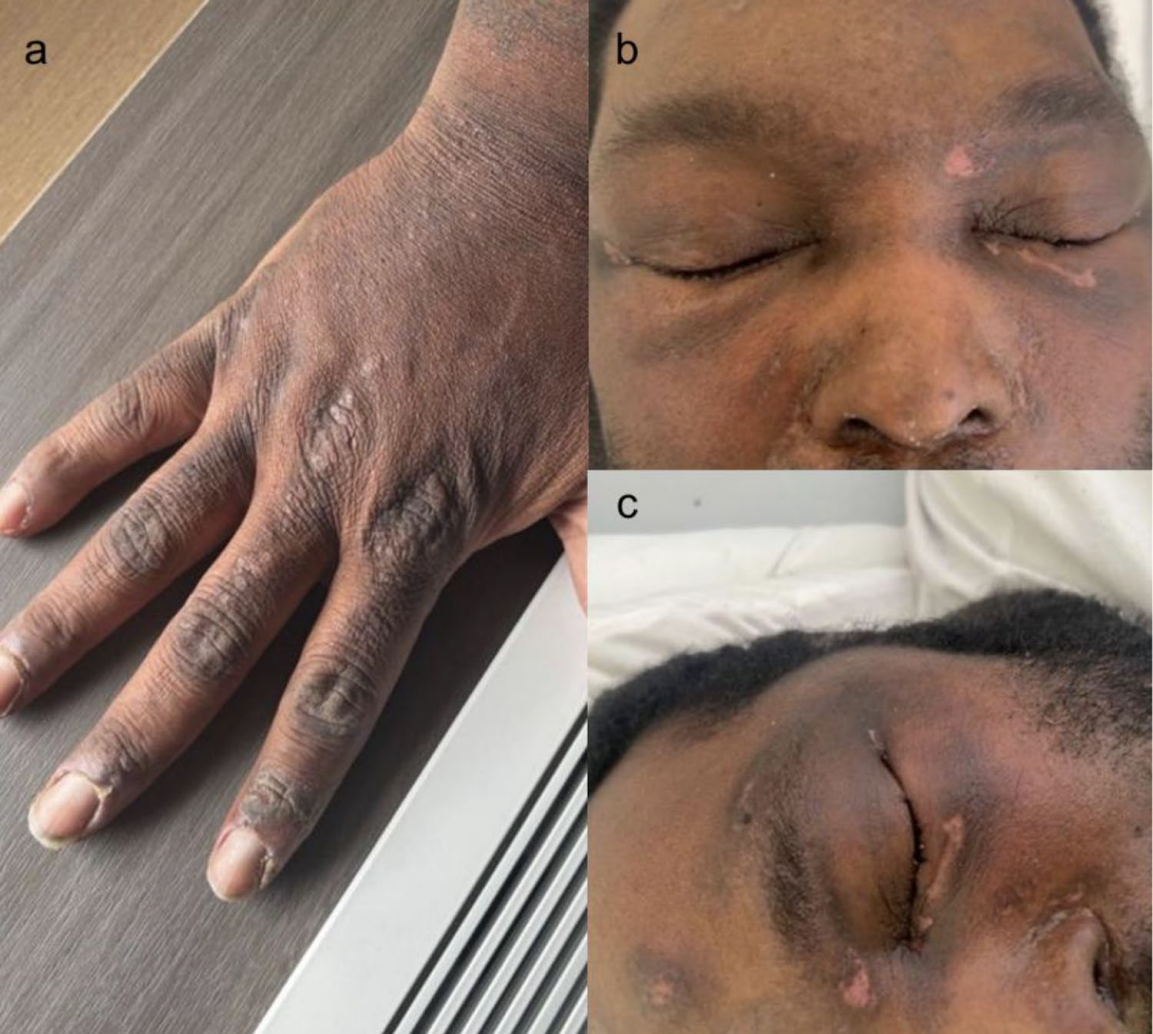
(a) Flat-topped, violaceous papules over the metacarpophalangeal and interphalangeal joints, most prominently on the second and third digits, consistent with Gottron’s papules. (b and c) Edema and subtle violaceous periorbital discoloration with a dull hue, suggestive of a heliotrope rash.

Shortly thereafter, the patient developed increasing proximal muscle weakness and myalgia, eventually becoming bedbound within 2 months of the initial dermatologic symptoms. Serum creatine kinase was markedly elevated (~3000 U/L), raising suspicion for dermatomyositis. On examination, violaceous-to-brown hyperpigmented periorbital discoloration with a dull hue, likely consistent with a heliotrope rash, was noted. Flat-topped, violaceous papules were also observed over the extensor surfaces of the metacarpophalangeal and interphalangeal joints, with extension to the extensor tendons, likely consistent with Gottron’s papules in darker skin phototypes (Figure 2). A comprehensive myositis panel revealed strong positivity for anti-TIF1-γ antibodies, which established the dermatomyositis diagnosis based on the EULAR/ACR criteria. After initial successful management of SJS/TEN with the aforementioned therapies and after establishing a diagnosis of dermatomyositis, the patient was treated using intravenous immunoglobulin therapy.

During hospitalization, a right axillary lymph node mass measuring 4.5 × 3.5 cm was discovered on chest computed tomography, which was shown to be intensely hypermetabolic on positron emission tomography scan. A core needle biopsy revealed a noncaseating granuloma, which was nondiagnostic. Subsequent excisional biopsy confirmed a diagnosis of DLBCL. Immunohistochemical staining was positive for CD20, BCL2, BCL6, and MUM1 (100%), with a Ki-67 proliferative index of 75%–80%. C-MYC expression was present in 80%–85% of malignant cells. The patient remains under multidisciplinary care for dermatomyositis and DLBCL, and is undergoing oncologic staging and treatment planning.

After the malignancy was diagnosed, the differential diagnosis included paraneoplastic pemphigus. However, skin biopsies showed no interface dermatitis, acantholysis, or dermal inflammatory infiltrate, making this unlikely. The absence of a drug trigger and the gradual, slow clinical progression are more consistent with SJS/TEN-like eruptions seen in autoimmune diseases such as lupus.^3^

We propose that the patient’s presentation reflects immune dysregulation triggered by paraneoplastic dermatomyositis or lymphoma, leading to clinically and histologically confirmed SJS/TEN development. This case highlights the need for further research into nondrug-induced triggers of SJS/TEN in autoimmune and malignant conditions.
